# Effects of nitrogen and vapour pressure deficit on phytomer growth and development in a C_4_ grass

**DOI:** 10.1093/aobpla/plw075

**Published:** 2016-11-03

**Authors:** Fang Yang, Xiao Ying Gong, Hai Tao Liu, Rudi Schäufele, Hans Schnyder

**Affiliations:** Lehrstuhl für Grünlandlehre, Department für Pflanzenwissenschaften, Technische Universität München, Alte Akademie 12, D-85354 Freising, Germany

**Keywords:** Coordination, developmental dynamics, elongation rate, growth duration, internode, leaf appearance, leaf blade, phyllochron, plant growth model, sheath

## Abstract

Phytomers are basic morphological units of plants. Knowledge of phytomer development is essential for understanding morphological plasticity, functional–structural modelling of plant growth and the usage of leaf characteristics to indicate growth conditions at the time of production (e.g. stable isotope signals). Yet, systematic analysis on the process of phytomer development is unavailable for wild or perennial C_4_ grasses. Also, effects of environmental factors, such as nitrogen nutrition or vapour pressure deficit (VPD), on coordination events of developmental processes of C_4_ grasses have not been studied. This study investigates phytomer growth and development in *Cleistogenes squarrosa*, a predominant C_4_ grass in the Eurasian steppe, grown at low (0.63 kPa) or high (1.58 kPa) VPD with low or high nitrogen supply in controlled environments. Elongation of phytomers on marked tillers was measured daily for 13 days. Then lengths of immature and mature phytomer components (blade, sheath and internode) of all phytomers were measured following dissection. Nitrogen nutrition and VPD had no effects on coordination of growth within and between phytomers: phytomer tips emerged when phytomers reached 26 % of their final length, coincident with the acceleration phase of its elongation; blade elongation stopped when phytomers reached ∼75 % of their final length and elongation of the preceding phytomer was confined to the internode. The relationship between fraction of final phytomer length and days after tip emergence for all treatments was well described by a sigmoidal function: *y* = 1/{1 + exp[(1.82 − *x*)/1.81]}. *C. squarrosa* exhibited little morphological plasticity at phytomer-level in response to nitrogen supply and VPD, but a clear increase in tillering under high N supply. Also, the invariant coordination of elongation within and between phytomers was a stable developmental feature, thus the quantitative coordination rules are applicable for predicting morphological development of *C. squarrosa* under contrasting levels of nitrogen nutrition or VPD.

## Abstract (precis)

The morphology of almost all grasses can be conceptualized as a hierarchical arrangement of subunits, termed phytomers. Therefore, knowledge of phytomer growth and development serves as a basis to elucidate the rhythm of grass growth. This study provides systematic analyses on the process of phytomer development of C. squarrosa, a perennial C4 grass. The invariant coordination of elongation within and between phytomers was a stable developmental feature across treatments, thus the quantitative coordination rules are applicable for predicting morphological development of C. squarrosa under contrasting levels of nitrogen nutrition or vapour pressure deficit.

## Introduction

The morphology of almost all grasses can be conceptualized as a hierarchical arrangement of subunits, termed phytomers, which are composed of a leaf blade, leaf sheath, node, internode and axillary bud in the leaf axil at the node between the leaf and the stem (Moore and Moser 1995). Phytomer development can be described as the succession of its components’ elongation which follows an invariable developmental sequence: the leaf blade starts to elongate first, followed by the sheath and the internode (for some species) (Fournier and Andrieu 1998). The size, number and spatial arrangement of phytomers determine the architectural organization of individual tillers (Briske 1991; Boe *et al.* 2000). Therefore, knowledge of phytomer growth and development serves as a basis to elucidate the rhythm of grass growth.

It has been repeatedly shown that the growth of phytomer components as well as the growth of successive phytomers is well coordinated and synchronized by certain events (e.g. leaf tip emergence or ligule emergence); namely, the dynamics of phytomer development are repeated in a certain sequence along the axis of a branch or tiller (Moore and Moser 1995; Boe *et al.* 2000; Boe and Casler 2005; Forster *et al.* 2007). Synchrony between emergence events and the dynamics of organ extension has been observed for species in which the internode does not elongate, such as C_3_ grasses in the vegetative stage (Skinner and Nelson 1995; Fournier *et al.* 2005), and for species with internode elongation, such as grasses during the generative (or reproductive) stages (Lafarge and Tardieu 2002; Zhu *et al.* 2014). The coordination rules underlying phytomer development form an important basis for plant growth models in different cereal crops, e.g. maize (Fournier and Andrieu 1998; Zhu *et al.* 2014), wheat (Evers *et al.* 2005; Vos *et al.* 2010) and barley (Buck-Sorlin *et al.* 2005 , 2008). The need to quantify the coordination of phytomer elongation, the timing of developmental events, growth duration, phyllochron and final phytomer number is critical for crop modelling (Evers *et al.* 2005). Furthermore, carbon and oxygen isotope compositions of leaf cellulose are potentially powerful tools for reconstruction of environmental conditions, e.g. oxygen isotope composition of leaf cellulose reflects mainly source water isotopic signal and evaporative demand (Helliker and Ehleringer 2002). Accordingly, oxygen isotope composition of leaf cellulose of *C.* *squarrosa* was shown to reflect VPD of the growth environment (Liu *et al.* 2016). For this type of studies, the predictability of phytomer development is essential for the dating of isotopic signals imprinted in plant material. In addition, the knowledge of phytomer development provides important insights into environmental influences on plant morphological and phenotypic plasticity (Boe *et al.* 2000). In wild (i.e. non-domesticated) or perennial C_4_ grasses phytomer development as well as the potential influence of environmental factors on coordination rules has not been studied, which impairs our understanding on the plasticity and predictability of morphological development.

In principle, environmental influences on plant development can result from changes in component elongation rate or from changes in component elongation duration (Zhu *et al.* 2014), or from the timing of development events. To explicitly elucidate how plants respond to varying environments, studies performed in the field and in controlled conditions have shown that leaf elongation rate in grasses is very sensitive to water supply (Passioura and Gardner 1990), vapour pressure deficit (VPD) (Ben Haj Salah and Tardieu 1996, 1997), and nutrient supply, especially of nitrogen (Volenec and Nelson 1983; Gastal and Nelson 1994). Nitrogen (N) deficiency slows leaf blade growth (Kavanová *et al.* 2008), decreases mature leaf size (Vos and van der Putten 1998) and reduces leaf appearance rate (Metay *et al.* 2014), but there is apparently little effect on leaf initiation (Ma *et al.* 1997). It has been reported that high evaporative demand reduces maize leaf elongation rate due to water deficit (Ben Haj Salah and Tardieu 1996, 1997). Accordingly, one may propose that phytomer growth and development in a wild grass with internode elongation in the vegetative state also responses to alterations of N and VPD supply. To know this phytomer morphological plasticity in different environments is of importance to broaden our understanding of plant life-history strategies.

Our objectives were to investigate phytomer growth and developmental characteristics of a perennial C_4_ grass (*Cleistogenes squarrosa*) and its morphological response to factorial combinations of nitrogen fertilization and VPD treatments. *C. squarrosa* is a perennial C_4_ grass and a co-dominant species in Inner Mongolia Grassland, a natural arid and semiarid ecosystem (Wang and Wang 2001; Bai *et al.* 2008). *C. squarrosa* has higher relative abundance in warm habitats such as sunny slopes (Gong *et al.* 2008), and it has been noted by authors that moderate to heavy grazing increased its relative abundance (Liang *et al.* 2002; Gong *et al.* 2008). Due to the dominance in many grassland types, especially degraded grasslands, *C. squarrosa* is considered as a key species for sustainable grassland and livestock management in this area (Liang *et al.* 2002). In Inner Mongolian grasslands, water and nitrogen are the primary limiting factors for plant growth (Bai *et al.* 2008); furthermore, interaction effects of N and water strongly influences physiology and morphology of dominant species (Gong *et al.* 2011). Moreover, soil properties exhibit strong spatial heterogeneity in nitrogen and other resources due to the inattentively managed grazing activities, e.g. grazing influences N turnover via faeces and urine redistribution (Giese *et al.* 2013). Thus, understanding phytomer development of *C. squarrosa* and its response to VPD and N nutrition, has important implications for improving productivity and forage quality in this grassland. In this work, we hypothesized, first, that phytomer development in *C. squarrosa* exhibits coordination rules similar to those found in other grasses, but with quantitatively different dynamics in phytomer development. Second, we predicted that the growth, characterized by both leaf and internode elongation, and the final length of phytomers are affected by N supply and VPD, mediated by the well-established effects of N and water stress on leaf elongation in other grasses (see above). Third, we predicted the constancy of coordination rules within and between phytomer growth across treatments. Aiming at quantitative descriptions on phytomer development of *C. squarrosa*, we assessed phytomer elongation both for visible growth (i.e. after emergence of the blade tip above the surrounding sheath of preceding phytomers) and invisible growth (i.e. before blade tip emergence), thus covering the whole elongation from (near) initiation to cessation of elongation.

## Methods

### Plant material and growth conditions

Seeds of *C. squarrosa* were collected in autumn 2010 and 2012 in the Xilin River watershed, China. Four seeds were sown in individual plastic tubes (5 cm diameter, 35 cm high) filled with quartz sand (0.3–0.8 mm diameter). Tubes were placed in free-draining plastic boxes (length: 77 cm, width: 57 cm, depth: 30 cm) with 164 tubes per box. Two boxes were placed in each of four growth chambers (Conviron PGR15, Conviron, Winnipeg, Canada). One week after sowing, plants were thinned to one plant per tube so that final density was 234 plants m^−^^2^. Except for nutrient supply and VPD, growth conditions were the same in all chambers: 16 h light period with a photosynthetic photon flux density of 800 µmol m^−^^2^ s^−^^1^ at canopy height during the light period, provided by cool-white fluorescent tubes, and constant air temperature of 25 °C throughout the light-dark cycle. CO_2_ concentration in the light period was kept constant between 380 and 390 μmol mol^−^^1^. Every eight hours, a modified Hoagland nutrient solution (see below) was supplied by an automatic irrigation system. The nutrient solution was completely renewed after 4 weeks, i.e. in the middle of experiments.

The study had a factorial design with two treatment factors, nitrogen supply and VPD, and each factor had two levels. Experiments were conducted in two separate runs with a low and high rate of N supply. All four chambers were supplied with a nutrient solution containing 7.5 mM N (N1) in the first run, and 22.5 mM (N2) in the second run, in the form of equimolar concentrations of calcium nitrate and potassium nitrate [Ca(NO_3_)_2_ and KNO_3_]. In each experimental run, two chambers were operated with a relative humidity of 80 % and the other two with 50 % relative humidity, yielding a VPD of 0.63 kPa (V1) and 1.58 kPa (V2), respectively. Thus, every combination of N supply and VPD (N1 V1, N1 V2, N2 V1 and N2 V2) was replicated in two growth chambers. The concentration of other nutrients was kept the same in both nutrient solutions: 1.0 mM MgSO_4_, 0.5 mM KH_2_PO_4_, 1 mM NaCl, 125 µM Fe-EDTA, 46 µM H_3_BO_3_, 9 µM MnSO_4_, 1 µM ZnSO_4_, 0.3 µM CuSO_4_ and 0.1 µM Na_2_MoO_4_.

### Monitoring and measurements of plant growth and calculation of phytomer elongation

Sixteen plants per treatment (eight plants per growth chamber) were tagged when the first true leaf had emerged. Ranks of leaves were counted acropetally starting from the base of the main tiller. Accordingly, the phytomer with the first formed leaf on the tiller, the first tagged leaf, was referred to as phytomer 1. We started to measure phytomer elongation on main tillers on the 24th day after the imbibition of seeds. At the start of elongation measurements, the main tiller had at least ten emerged leaves. Elongation measurements lasted until the 37th day after imbibition. This corresponds to a thermal time of 925 degree days, close to the thermal time of the main vegetation period for *C. squarrosa* in Inner Mongolia (Liang *et al.* 2002). This also ensured that a sufficiently high number of phytomers completed their growth during the experiment. Each day, the distance between the tip of a phytomer and the next older visible ligule, and also the distance between successive visible ligules was measured to the nearest 0.5 mm. Our preliminary data showed that sheath elongation stopped with the emergence of the ligule (data not shown). Thus, phytomer elongation was calculated as the change of distance from phytomer tip to the next older visible ligule. A phytomer was considered fully expanded when the distance from its tip to the next older visible ligule had ceased to change. In most cases, tips of phytomers 12 and 13 appeared (leaf tips emerged) and terminated elongation during the observation period between 24 and 37 d after imbibition. These data were used to calculate ‘visible growth duration’ and the visible time course of phytomer elongation. Phytomer tip emergence was defined as the moment a blade tip had grown past the highest visible ligule of the preceding phytomers. However, as elongation measurements were performed only once a day, a phytomer tip could have emerged before our first observation on that phytomer. Therefore, the time lag between tip emergence and our first observation on that phytomer was estimated as the distance between the tip of the phytomer in question and the next older visible ligule divided by the elongation rate measured just following tip emergence; and this time lag was accounted for in the calculation of days after tip emergence. The phyllochron was calculated as the time interval between the appearances of successive phytomers. The number of tillers formed at the base of the shoot was also recorded.

### Measurements on length of phytomers and their components

**Figure 1:**
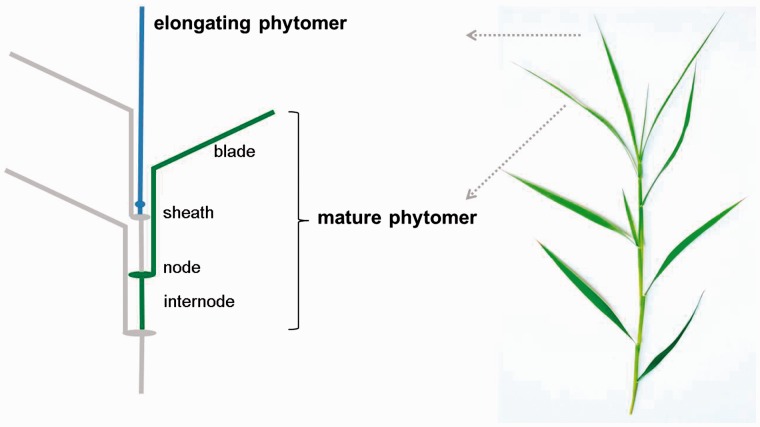
Schematic of a mature phytomer and its components, and arrangement of phytomers along a tiller of *C. squarrosa*. The ligule (or collar; data not shown) marks the blade-sheath junction, and the node forms the site of insertion of the leaf on the stem. Immature, growing phytomers are situated at the tip of the tillers (note: for simplicity, the scheme depicts only two growing phytomers, but up to five concurrently expanding phytomers in different developmental stages are found along the distal part of tiller axis upon dissection).

At the end of each experiment, all main tillers (16 main tillers from 16 plants per treatment) on which elongation rate had been measured were sampled and dissected into individual phytomers. On these dissected phytomers, the lengths of blades, sheaths and internodes were measured to the nearest 0.5 mm, and phytomer length was obtained as the sum of the length of its components. Thus, destructive measurements provided the actual length of blades, sheaths, internodes and the total length of successive phytomers. Dissected tillers included fully expanded phytomers, emerged (i.e. visibly growing) phytomers and unemerged (invisibly growing) phytomers whose tips had not yet emerged. A scheme of phytomer arrangement along successive nodes on one tiller is presented in Fig. 1. Within a tiller, all phytomers are arranged along an axis. The basipetal succession of phytomers corresponds to a gradient of increasing phytomer age. Each phytomer is composed of a leaf blade, a leaf sheath, a node and an internode. Phytomer elongation starts with the (tip of the) blade and successively passes over to elongation of the sheath and internode. The sheath of a given phytomer enclosed the internode of the next younger phytomer and a part of its sheath. In the dissection, only phytomers with a length >4 mm were taken into consideration excluding the shoot apex with shorter phytomers and primordia.

### Assessing coordination within a phytomer and between phytomers

The length of successive, fully expanded phytomers (obtained from destructive measurements) was used to determine ontogenetic changes in the final length of phytomers, i.e. the relationship between final phytomer length and phytomer rank. By extrapolating this relationship, the final length of still growing phytomers (immature ones) was predicted. That prediction was performed for each individual tiller, using the data from that same tiller. The fraction of final phytomer length (*f*_L_) for immature phytomers was calculated as:

(1) $$f_{L}\;=\;L_{a}/L_{P}$$

where *L*_a_ is the actual phytomer length at a given time, *L*_p_ is predicted final phytomer length.

Accordingly, the fraction of final phytomer length is 1 for a fully expanded phytomer. In an analogous manner, the fractional contribution of components (blade, sheath, internode) to final phytomer length was calculated as the ratios of actual blade-, sheath- and internode-length to predicted final phytomer length.

The coordination of elongation within a phytomer was assessed by plotting the fractional contribution of components (blade or sheath or internode) to final phytomer length against the fractions of final phytomer length. The coordination of elongation between phytomers was assessed by analysing the relationship between fractions of final phytomer length of successive phytomers. The normalized length of components and phytomers was used to compensate for heterogeneity in plant size, which is higher for wild plants than for genetically much more uniform cultivars.

### Constructing the time course of phytomer development

A young phytomer grows within the sheath tube of the preceding phytomer before tip emergence. Therefore, the complete time course of the development of a phytomer includes a visible phase (tip emergence − full expansion) and an invisible phase (initiation − tip emergence). These time courses were all established by plotting the fraction of final phytomer length against their ages (days after tip emergence).

The visible time course of phytomer development was established using the data of phytomers (ranks 12 and 13) on which the elongation rate had been monitored from tip emergence to full expansion. From the dissection of plants we obtained the final length of those phytomers, and the actual length of those phytomers on each day during expansion was calculated retrospectively using the measured daily elongation rate. Thus, the fraction of final phytomer length of those phytomers was calculated using Equation (1) (using the actual measured final length as denominator) for each day from tip emergence to full expansion.

The initial phase of phytomer development was established using the data of younger phytomers which were not fully elongated (immature) at the time of dissection. For these phytomers, their fractions of final phytomer length were determined using Equation (1). As their ages (the corresponding days after their tip emergence) were not known, these had to be estimated. In each tiller, an immature phytomer was selected that had reached 60–80 % of its final length (typically the second oldest immature phytomer). Using the visible time course of phytomer development (see previous paragraph) the time after tip emergence of this particular phytomer was estimated. This time minus the respective number of phyllochrons gave the time after tip emergence for each phytomer younger than the selected one on this tiller. We plotted the fraction of final phytomer length against the calculated age of those immature phytomers to obtain the initial phase of phytomer development that included the invisible phase and overlapped with a small range of the visible phase of phytomer development. The construction of the initial phase of phytomer development was done separately for each treatment, using the treatment-specific data. The visible time course and initial phases of phytomer development were then combined to obtain the complete time course (i.e. the fraction of final phytomer length vs. days before/after tip emergence).

### Statistical analyses

The effects of N supply (low and high N: N1 and N2), VPD (low and high VPD: V1 and V2) and their interaction on phytomer growth parameters were analysed by two-way analysis of variance (ANOVA) using R (R Core Team, version 3.1.2, 2012). There were 16 replicates (16 main tillers: eight tillers per growth chamber) in each treatment. For parameters that were measured on several ranks of phytomers of a tiller, i.e. phyllochron, visible growth duration, maximal phytomer elongation rate and final phytomer length, the mean value of all phytomers on that tiller was used in the statistical tests. For repeatedly observed parameters, e.g. the number of visibly growing phytomers, the total number of phytomers, and the number of tillers, the mean values of repeated observations on each tiller were used in the statistical tests. The data were analysed with a linear model using the generalized least squares method. This was implemented with the generalized least squares (gls) function by maximizing the restricted log-likelihood (REML) method in the ‘nlme’ package (Pinheiro *et al.* 2014), and the chamber effect was also included in the model. None of the analyses yielded a significant chamber effect. For the developmental pattern of phytomers, 95 % confidence bands of regressions were fitted using Sigmaplot (Systat Software, San Jose, CA) and used to test for treatment effects.

## Results

### Phytomer growth and developmental parameters

**Figure 2:**
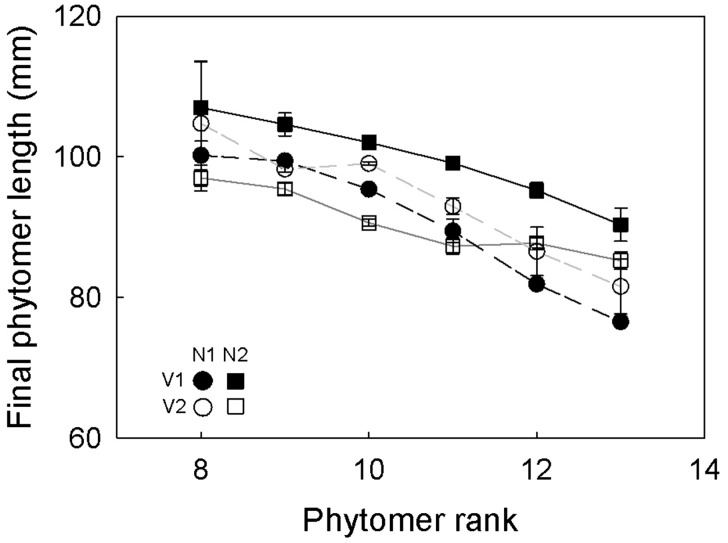
Final (mature) length of phytomers along the axis of the main tiller of *C. squarrosa* under contrasting nitrogen fertilizer and VPD treatments. Phytomer rank was counted acropetally from the base of the tiller. Error bars indicate SE (*n* = 16).

Averaged over all experiments (Table 1), the number of emerged (non-enclosed) phytomers that were growing simultaneously averaged 3.9 per main tiller. The average number of phytomers per main tiller observed during the 24th–37th day after imbibition was ∼14. The phyllochron averaged 2.3 days, and the duration of visible growth (i.e. the time span from tip emergence to cessation of elongation) was about 11 days. Mean number of tillers was 21 and mean phytomer length was 93 mm (Fig. 2). In all treatments, final phytomer length decreased by about 20 % between phytomer rank 8 and rank 13, the last phytomer which terminated its elongation during the observation period (Fig. 2).

**Table 1:** Growth and developmental parameters of *C.squarrosa* under contrasting N fertilizer and VPD treatments and the results of two-way ANOVA for each parameter.

Parameters	Treatment				*F* value			
	N1 V1	N1 V2	N2 V1	N2 V2	N	VPD	N × VPD	Df
Number of visibly growing phytomers (tiller^−1^)	3.6 ± 0.1	3.8 ± 0.1	4.2 ± 0.1	3.8 ± 0.1	6.083*	2.257	8.276*	58
Number of phytomers (tiller^−1^)	13.4 ± 0.2	13.3 ± 0.2	14.5 ± 0.2	13.7 ± 0.2	8.073*	4.035	1.938	59
Phyllochron (days leaf^−1^)	2.4 ± 0.1	2.4 ± 0.0	2.1 ± 0.0	2.4 ± 0.1	8.627*	2.663	4.734*	60
Visible growth duration (days)	11.0 ± 0.5	11.8 ± 0.2	10.6 ± 0.2	10.6 ± 0.3	12.131*	4.604*	3.498	57
Number of tillers	19.7 ± 1.8	17.6 ± 1.0	24.0 ± 1.1	21.7 ± 1.6	7.754*	2.005	0.003	60

A low or high N fertilizer supply (N1 or N2) was combined with low or high VPD (V1 or V2). Values are means ± SE (*n* = 16). The number of visibly growing phytomers on a tiller, the number of phytomers per main tiller, and the number of tillers at the base of the plant are mean values for the period between 24 and 37 days after imbibition; phyllochron was measured on phytomer ranks 7–16; visible growth duration, the period from tip emergence to the cessation of elongation, was measured on ranks 12–13. Asterisk indicates significant effect (*P* < 0.05). Df is the degree of freedom for the denominator in F and the degree of freedom for the numerator is 1 in all cases.

Effects of VPD and N fertilizer supply or their interactions were relatively small or non-significant, except for the effect of N fertilizer supply where high N increased the number of tillers by 23 % relative to low N (averaged over VPD levels) (Table 1). Also, averaged over VPD levels, high N increased the number of phytomers per tiller by 6 %, and shortened visible growth duration by 7 %. Averaged over N levels, high VPD increased visible growth duration by 4 % relative to low VPD. All other parameters of phytomer growth and development were influenced by the interaction of nitrogen fertilizer supply and VPD. Thus, high N increased the number of visibly growing phytomers per tiller by 17 % and phytomer length by 8 %, and shortened the phyllochron by 13 % at low VPD, but not at high VPD.

### Coordination within a phytomer

**Figure 3:**
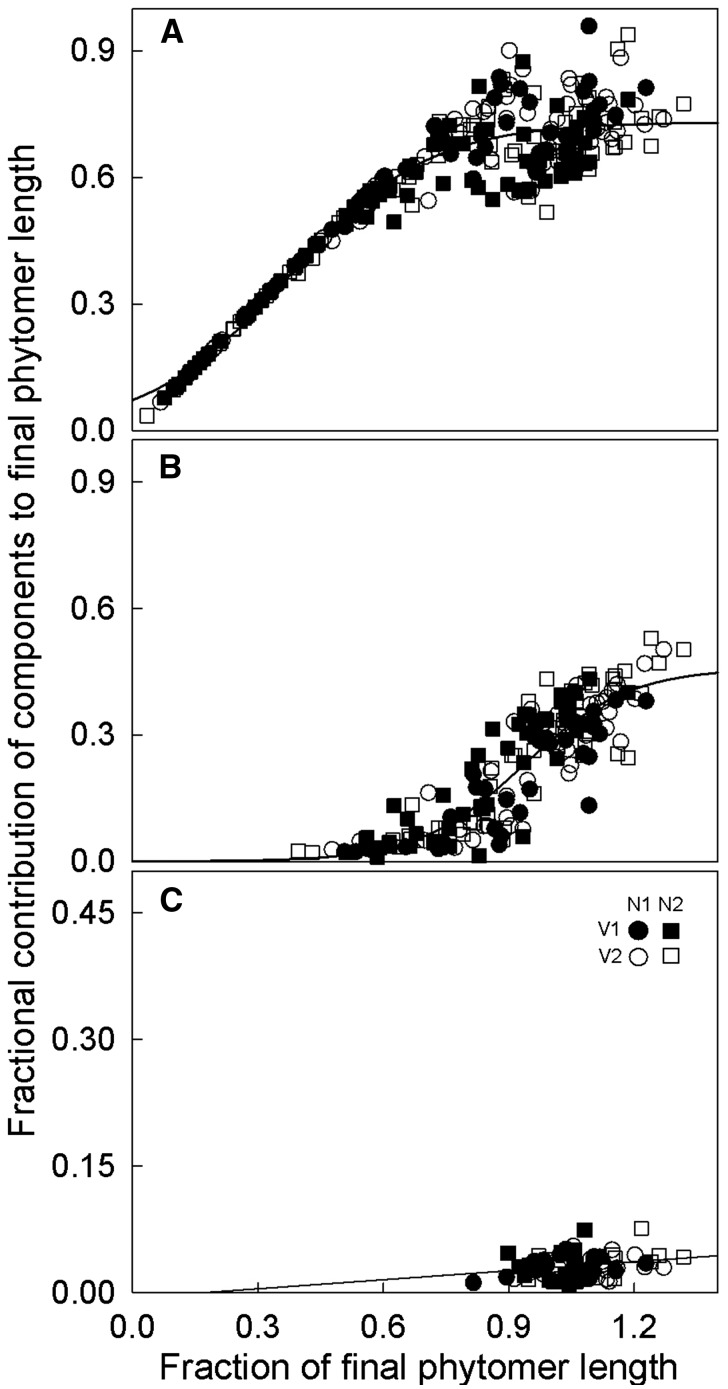
Fractional contributions of phytomer components to the final length of phytomers at successive stages of phytomer elongation of *C. squarrosa* under contrasting N fertilizer and VPD treatments. Panel A, blade; panel B, sheath; Panel C, internode. The solid lines were fitted to the data of all treatments. Stage of elongation is given by the fraction of final phytomer length, estimated as the ratio of the actual phytomer length to the predicted final phytomer length (see ‘Material and Methods’ section). All data were obtained from destructive measurements. Each point corresponds to a single measurement.

The contributions of phytomer components (blade, sheath and internode) to final phytomer length in expanding phytomers did not show treatment effects [Fig. 3A–C; **see Supporting Information—Fig. S1** for confidence intervals of the different treatments], so data of all treatments were pooled for further analysis. Solid lines in Fig. 3A–C denote regressions with *y*  =  0.73/{1 + exp[(0.37− *x*)/0.17]} (*R*^2 ^ = ^ ^0.90; residual standard error  =  0.06) for blade (A); *y*  =  0.46/{1 + exp[(0.94 − *x*)/0.13]} (*R*^2 ^ = ^ ^0.78; residual standard error  =  0.07) for sheath (B); *y*  =  0.04*x – 0.0063 (*R*^2 ^ = ^ ^0.06) for internode (C). At the time of the final sampling, five phytomers, including emerged and unemerged phytomers, were expanding simultaneously on a tiller **[see Supporting Information—Fig. S1]**. The relationship between the contribution of components to final phytomer length and fractional final phytomer length showed a gradual transition between different phytomer ranks (from just after initiation of rapid expansion to near-fully expanded). Hence, the phytomers of different ranks were treated as representing one phytomer in different developmental stages. Within a phytomer, initial expansion was exclusively due to blade elongation (Fig. 3A). Transition between blade and sheath elongation was a relatively sudden event. Sheathes were first measured when phytomers had reached approx. 50 % of their final length (Fig. 3B); moreover, initial sheath elongation was slow. When blade length had reached 66 ± 1 % (SE) of final phytomer length, blades ceased to elongate and rapid sheath elongation began. As the phytomer had attained 75 % of its final length by that time, initial sheath elongation must have contributed 9 % to final phytomer length. Internode elongation started when the phytomer had reached approx. 80 % of its final length (Fig. 3C). Transition between sheath and internode elongation also appeared to be a relatively sudden event, as internodes significantly contributed to phytomer elongation only when sheath elongation was nearly terminated.

### Coordination between successive phytomers

**Figure 4:**
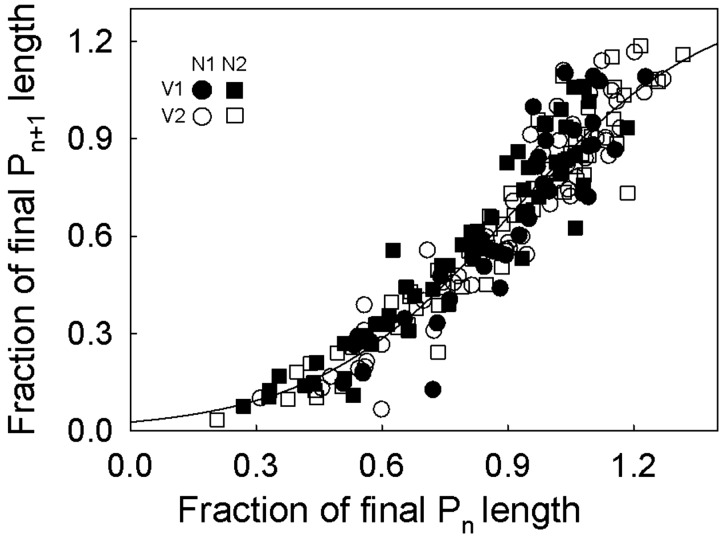
Relationships between the lengths of two successive immature (non-fully elongated) phytomers of *C. squarrosa* under contrasting N fertilizer and VPD treatments. The solid line was fitted to the data of all treatments. P_n_ refers to any given immature phytomer and P_n + 1_ to the corresponding next younger phytomer. All data were obtained from destructive measurements. Each point corresponds to a single measurement.

Similar to the relationships within a phytomer, the coordination of development of successive phytomers was not affected by treatments (Fig. 4; [**see Supporting Information—Fig. S2** for confidence intervals of parameters and phytomer rank]). A sigmoidal function (solid line in A) was fitted to the combined data of all treatments: *y*  =  1.34/{1 + exp[(0.91 − *x*)/0.24]} (*R*^2 ^ = ^ ^0.90; residual standard error  =  0.1). A given phytomer (P_n + 1_) did not elongate significantly until the preceding phytomer (P_n_) had reached approx. 25 % of its final length. Then, P_n + 1_ elongated more slowly than P_n_ until P_n_ had reached approx. 50 % of final phytomer length. When the younger phytomer (P_n + 1_) had elongated to approx. 20 % ± 3 % (SE) of its final length, both phytomers elongated at nearly the same rate until P_n_ was near-fully elongated (defined here as 99 % of the final length). At that time, P_n + 1_ had reached 79 ± 2 % (SE) of its final length, which corresponded to the length at which blade growth stopped (see previous paragraph).

### Kinetics of a phytomer development

**Figure 5:**
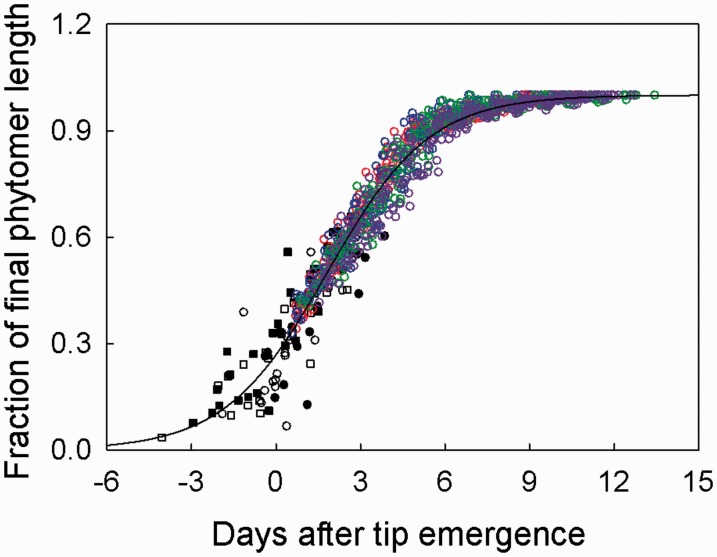
Time course of the fraction of final phytomer length in *C. squarrosa* under contrasting N fertilizer and VPD treatments. Coloured circles represent the visible phase of the time course of phytomer development (see Materials and Methods): green circles, N1 V1; purple circles, N1 V2; red circles, N2 V1; blue circles, N2 V2. Black and white symbols represent the initial phase of phytomer development based on predictions of age and the final length: closed circles, N1 V1; open circles, N1 V2; closed squares, N2 V1; open squares, N2 V2. The curve denotes the two-parameter sigmoidal function for all data: *y* = 1/{1 + exp[(1.82− *x*)/1.81]}, (*R*^2 ^=^ ^0.96, residual standard error = 0.04).

For a small number of phytomers (mainly ranks 12 and 13), the observation period of phytomer elongation covered the complete visible phase of phytomer development, which began with the appearance of the leaf tip and ended with the cessation of internode elongation. In all treatments, the visible time course of phytomer elongation followed a sigmoidal pattern (coloured symbols in Fig. 5). This relationship was nearly identical for the four treatments [**see Supporting Information—Fig. S3 and Table S1** for confidence intervals of parameters]. The curve denotes the two-parameter sigmoidal function for all data: *y*  =  1/{1 + exp[(1.82− *x*)/1.81]} (*R*^2 ^ = ^ ^0.96; residual standard error  =  0.04). At the time of tip emergence, phytomers had attained approx. 26 % of their final lengths in all treatments. After tip emergence, phytomer length increased linearly with time until reaching approx. 85 % of final length, approx. 5 days after emergence. Thereafter, phytomer elongation decreased, but continued for another 6 days. The visible period of phytomer elongation (defined here as 99 % of the final length) terminated at about 11 days after tip emergence.

The reconstruction of the initial phase of phytomer development using the immature phytomers was based on the notion that the difference in age between successive phytomers corresponds to the phyllochron. Thus, data on immature phytomers and phytomers which finished elongation during the observation period could be combined to establish the complete time course of phytomer elongation from 3.5 % of final phytomer length to cessation of elongation (Fig. 5). Data of immature phytomers (black and white symbols in Fig. 5), for which the age had been estimated, and data of phytomers, for which the age had been directly measured (coloured symbols in Fig. 5) fit a two-parameter sigmoidal function: *y*  =  1/{1 + exp[(*x*_0_ − *x*)/b]}, with *y* denoting the fraction of final phytomer length, *x* days after tip emergence, *b* the slope of the curve and *x*_0_ the time when phytomer growth rate was maximum. There was no significant treatment effect on the complete time course of phytomer expansion **[see Supporting Information—Fig. S4 and Table S2]**. For example, phytomers reached maximal growth rate at about 2 days after tip emergence in all treatments. Starting from 3.5 % of final length, the estimated time until tip emergence was approx. 4 days. The phase of near-linear maximum elongation began at about 1 day before appearance of the leaf tip when the phytomer had reached approx. 20 % of final phytomer length, and ended about 4.5 days after tip appearance when the phytomer had reached approx. 85 % of final length. These phases corresponded with the phases of blade and sheath elongation (Fig. 3A and B). The last elongation phase was associated with internode elongation, which occurred at considerably lower rates (Fig. 3C).

## Discussion

Our study showed little morphological plasticity of *C. squarrosa* at phytomer-level in response to nitrogen supply and VPD, and the main variation in morphology was observed in tillering across N levels. The coordination of growth within a phytomer and between phytomers of *C. squarrosa* was virtually unaffected by N nutrition and VPD. Although plant development is highly sensitive to environmental conditions (Moulia *et al.* 1999), to the best of our knowledge, growth coordination in *Gramineae* has so far only been studied in relation to temperature (most often expressed as thermal time (Lafarge and Tardieu 2002; Fournier *et al.* 2005). In field studies, where VPD inevitably varies over the growing period, the timing of key events of phytomer development was fully explained by thermal time, which indicates that VPD had no effect on coordination (Lafarge and Tardieu 2002; Fournier *et al.* 2005).

The constancy of coordination across treatments should be related to the regularity of trigger events. The emergence of the blade tip is recognized as one trigger event for coordination, although the exact signalling involved is not clear yet (Fournier *et al.* 2005; Zhu *et al.* 2014). Sheath tube length affects the development of a young phytomer (Wilson and Laidlaw 1985; Casey *et al.* 1999), as cell production of the blade is thought to be regulated by the light signal received by the emerging blade tip. In this study, the relationship between blade length and previous sheath length showed no treatment response **[see Supporting Information—Fig. S5 and Table S3]**. Moreover, at the time of tip emergence, phytomers had reached on average 26 % of their final length with very little variation between treatments. Hence, if N nutrition and VPD treatment do not affect the relationship of blade and sheath tube length, coordination rules can be generalized for *C. squarrosa* across nitrogen and VPD treatments.

### Coordination of phytomer development

The elongation of a phytomer of *C. squarrosa* followed a sigmoidal pattern similar to that found in other grasses like maize (Fournier and Andrieu 2000), wheat and tall fescue (Fournier *et al.* 2005). It has been shown that, for wheat and tall fescue (Fournier *et al.* 2005), the elongation of a leaf can be subdivided into four phases. While in this study, the initial phase of phytomer elongation was not captured, the acceleration phase, the quasi-linear phase and the elongation-decay phase were also apparent in *C. squarrosa*. The transition between the acceleration phase and the quasi-linear phase occurred 1 day before the tip emergence. Thus, the first phase of visible elongation corresponded to the quasi-linear phase of phytomer elongation, when phytomer elongation was still exclusively due to blade elongation. Maximum elongation rates were observed at 2 days after tip emergence, when phytomers had reached 52 % of their final length. This corresponded to blade elongation, as final blade length contributed 63 % to final phytomer length. Although elongation rates during the visible growth phase were continuous without a significant break, transition between blade and sheath growth must have occurred suddenly, as significant sheaths were only observed when blades were (nearly) fully elongated as revealed also in the destructive harvest. Research on a variety of grass species has shown that significant internode elongation is synchronized with the end of elongation of the associated sheath in maize (Hesketh *et al.* 1988), in sorghum (Lafarge *et al.* 1998) and in barley (Kirby *et al.* 1994). Here, the final gradual fading out of phytomer elongation was mainly associated with internode elongation. Hence, elongation rates for the internode were significantly lower than elongation rates of blade and sheath. This difference is associated with the activity of different meristems responsible for leaf (blade *plus* sheath) and internode growth.

Elongation of successive phytomers was also tightly coordinated: thus, the acceleration phase of the elongation of a young phytomer commenced, when the preceding phytomer had reached approx. 25 % of its final length, i.e. when the tip of the preceding phytomer emerged. This may also indicate a signal transduction within a tiller based on tip emergence, as some studies discussed the regulation of shoot development by hormonal signals (Tamas 1995; Horvath *et al.* 2003; Schmitz and Theres 2005). When a phytomer terminated elongation, the blade of the next younger phytomer was fully expanded and its internode started to elongate.

Due to the tight coordination in phytomer growth across N and VPD levels, the time course of phytomer development was describable using one single equation. The duration of elongation from the time when the phytomer had reached 3.5 % of final length until the cessation of elongation (99 % of final length) was approx. 13 d, which corresponds to 185 degree days—approx. half of the thermal time necessary to complete phytomer elongation in *Sorghum bicolor* (assuming the same base temperature of 10.8 °C; Lafarge and Tardieu 2002), a C_4_ annual grass and crop species. Moreover, the duration of the linear elongation phase was about 6 days, i.e. 85 degree days in our study, which is again approx. half of that reported for *S. bicolor* (Lafarge and Tardieu 2002).

### Effects of VPD and N supply on plant morphology

The overall effects of VPD and N supply on phytomer development were small. N fertilization supply slightly increased the number of visibly growing phytomers and phytomer length, and shortened the phyllochron at low VPD but not at high VPD. Positive effects of nitrogen fertilization and low VPD on leaf elongation are well established (MacAdam *et al.* 1989; Gastal and Nelson 1994; Ben Haj Salah and Tardieu 1996; Vos and van der Putten 1998; Tardieu *et al.* 2000; Kavanová *et al.* 2008). Although high VPD clearly increased the leaf-level transpiration rate and decreased stomatal conductance in the same experiment (Gong *et al.* 2016), the non-significant VPD effects on phytomer growth parameters may be due to the non-limiting water supply in our experiment, i.e. the supplementation of nutrient solution with a frequency of three times per day. Grass species were shown to be able to adjust root hydraulic conductance in response to VPD (Ocheltree *et al.* 2014) to maintain a high leaf water potential if water supply is sufficient. The main variation in plant morphology was observed in tiller development and growth, namely, the number of tillers was significantly increased by 23 % by N supply. These results are in line with the findings that whole-plant relative growth rate of *C.* *squarrosa* increased following N addition but was not affected by VPD (Liu *et al.* 2016). A positive effect of N supply on the tillering of grasses is a common response (Longnecker *et al.* 1993; Baethgen *et al.* 1995; McKenzie 1998), as N fertilizer promotes the outgrowth of axillary meristems to produce more tillers (McSteen 2009). For barley, it has been shown that wild varieties exhibit a similar affinity to N as crop cultivars (Bloom 1985). Thus, the low N treatment exerted only a moderate limitation, which did not limit the development of individual phytomers. However, the reduced phyllochron in the high N treatment might be responsible for the positive effect on tillering, as it increases the number of axillary meristems.

## Conclusions

Our results revealed a strong coordination of elongation both within a phytomer and between successive phytomers on a tiller of *C. squarrosa*, a wild, perennial C_4_ grass. The coordination in phytomer development was virtually unaffected by nitrogen nutrition and VPD, which indicates that tiller development of *C. squarrosa* can be captured using general quantitative coordination rules across VPD or nitrogen nutrient levels. On the other hand, a clear response in tillering to N addition was observed. Similarly, a field study showed that both tiller weight and tiller density responded to N and water supply in this grassland ecosystem (Gong *et al.* 2015). These results emphasize the importance of understanding tillering kinetics in modelling the whole shoot morphological development of *C. squarrosa*. Tip emergence, a key event, may have a crucial role in signal transduction to coordinate phytomer elongation. Accordingly, the time course of phytomer development was established using observations on visibly growing phytomers, applying a sigmoidal function. This quantitative discription of phytomer development potentially can be used for: functional–structural modelling, analysing morphological plasticity, and indicating environmental factors using leaf isotopic signals of *C. squarrosa*.

## Sources of Funding

This research was funded by the Deutsche Forschungsgemeinschaft (DFG SCHN 557/7-1). F. Y. and H. T. L were supported by the Chinese Scholarship Council (CSC).

## Contributions by the Authors

H.S., R.S., F.Y., and X.Y.G. designed and planned the research. F.Y. performed the measurements, analyzed the data and wrote a first draft. All authors contributed to the discussion of the data, writing and revision.

## Conflict of Interest Statement

None declared.

## Supplementary Material

Supplementary Data

Supplementary Data
