# Supplementary material for: Effects of nitrogen and vapour pressure deficit on phytomer growth and development in a C4 grass
Source: AoB Plants. 2016 Nov 3;8:plw075. doi: 10.1093/aobpla/plw075 (PMC5206350; doi:10.1093/aobpla/plw075)
Supplement: Supplementary Data [file supp_8_plw075_index.html]

Effects of nitrogen and vapour pressure deficit on phytomer growth and development in a C4 grass — Effects of nitrogen and vapour pressure deficit on phytomer growth and development in a C4 grass — Supplementary Data 

# Effects of nitrogen and vapour pressure deficit on phytomer growth and development in a C4 grass

## Supplementary Data

files

- Supplementary Data - docx file
