# Supplementary material for: Effects of nitrogen and vapour pressure deficit on phytomer growth and development in a C4 grass
Source: AoB Plants. 2016 Nov 3;8:plw075. doi: 10.1093/aobpla/plw075 (PMC5206350; doi:10.1093/aobpla/plw075)
Supplement: Supplementary Data [file supp_plw075_Supporting_information.docx]

Fig. S1. Comparison of fractional contributions of blade (Panle A) or sheath (Panle B) to the total length of phytomers at successive stages of phytomer elongation under contrasting N fertilizer and VPD treatments. A low or high N fertilizer supply (N1 or N2) was combined with low or high VPD (V1 or V2). Panel A and B specify the data according to the treatments: closed circles with green lines, N1 V1; opened circles with purple lines, N1 V2; closed squares with red lines, N2 V1; opened squares with blue lines, N2 V2. Panel C (blade) and D (sheath) show the same data, but depict different phytomer ranks (open triangles, P_S_; open squares, P_S+1_; crosses, P_S+2_; open diamonds, P_S+3_; open circles, P_S+4_). P_S_ indicates the phytomer that bore the youngest emerged ligule (youngest near-fully expanded phytomer with ligule visible above the surrounding leaf sheath of the next older phytomer); P_S+1_, P_S+2,_ P_S+3_ and P_S+4_ refer to progressively younger phytomers. All data were obtained from destructive measurements. Each point corresponds to a single measurement on one phytomer. Lines denote the 95% confidence bands of the fitting function.

Fig. S2. Comparison of relationships between the lengths of two successive immature (non-fully-elongated) phytomers under contrasting N fertilizer and VPD treatments. A low or high N fertilizer supply (N1 or N2) was combined with low or high VPD (V1 or V2). Panel A differentiates treatments: closed circles with green lines, N1 V1; opened circles with purple lines, N1 V2; closed squares with red lines, N2 V1; opened squares with blue lines, N2 V2. Panel B shows the same data, but depicts different phytomer ranks: open squares, P_S+1_ vs. P_S_; crosses, P_S+2_ vs. P_S+1_; open diamonds, P_S+3_ vs. P_S+2_; open circles, P_S+4_ vs. P_S+3_. P_S_ indicates the phytomer that bore the youngest emerged ligule (youngest near-fully elongated phytomer with ligule visible above the surrounding leaf sheath of the next older phytomer); P_S+1_, P_S+2,_ P_S+3_ and P_S+4_ refer to progressively younger phytomers. Each point corresponds to a single measurement. Lines denote the 95% confidence bands of the fitting function.

Fig. S3. Comparison of visible time courses of the fraction of final phytomer length under contrasting N fertilizer and VPD treatments *.*A low or high N fertilizer supply (N1 or N2) was combined with low or high VPD (V1 or V2). These visible time courses were established using data of phytomers on which the phytomer elongation has been measured from the time of leaf tip emergence to full elongation. Tip emergence was defined as the moment a leaf blade tip had grown past the highest visible ligule of the preceding phytomers. Sigmoidal regressions were fitted to each treatment. Lines denote the 95% confidence bands of the fitting function. Green circles with green lines, N1 V1, y = 1/{1 + exp [(1.87 − x)/1.73]} (R^2^ = 0.98, n = 27, residual standard error = 0.03); purple circles with purple lines, N1 V2, y = 1/{1 + exp[(1.94 − x)/1.90]} (R^2^ = 0.97, n = 31, residual standard error = 0.03); red squares with red lines, N2 V1, y = 1/{1 + exp[(1.69 − x)/1.71]} (R^2^ = 0.99, n = 25, residual standard error = 0.03); blue squares with blue lines, N2 V2, y = 1/{1 + exp[(1.73 − x)/1.82]} (R^2^ = 0.98, n = 26, residual standard error = 0.03). A sigmoidal regression was fitted for all data: y = 1/{1 + exp[(1.79 − x)/1.81]} (R^2^ = 0.96, n = 109, residual standard error = 0.03).

Fig. S4. Comparison of the complete time courses of the fraction of final phytomer length *C. squarrosa* under contrasting N fertilizer and VPD treatments. A low or high N fertilizer supply (N1 or N2) was combined with low or high VPD (V1 or V2). Coloured circles represent the visible phase of the time course of phytomer development: green circles, N1 V1; purple circles, N1 V2; red circles, N2 V1; blue circles, N2 V2. Black and white symbols represent the initial phase of phytomer development based on predictions of age and the final length: closed circles, N1 V1; open circles, N1 V2; closed squares, N2 V1; open squares, N2 V2. Tip emergence was defined as the moment a leaf blade tip had grown past the highest visible ligule of the preceding phytomers. Sigmoidal functions were fitted to the complete time courses of each treatment. Lines denote the 95% confidence bands of the fitting function. Green circles with green lines, N1 V1, y = 1/{1 + exp[(1.97 − x)/1.69]} (R^2^ = 0.96, residual standard error = 0.04); purple circles with purple lines, N1 V2, y = 1/{1 + exp[(2.02 − x)/1.84]} (R^2^ = 0.96, residual standard error = 0.04); red squares with red lines, N2 V1, y = 1/{1 + exp[(1.61 − x)/1.79]} (R^2^ = 0.98, residual standard error = 0.03); blue squares with blue lines, N2 V2, y = 1/{1 + exp[(1.75 − x)/1.82]} (R^2^ = 0.97, residual standard error = 0.04).

Fig. S5. Relationships between the length of a blade and the length of its preceding sheath (i.e. length of blade Nr. n+1 vs. length of sheath Nr. n) in mature phytomers of *C. squarrosa* under contrasting N fertilizer and VPD treatments. A low or high N fertilizer supply (N1 or N2) was combined with low or high VPD (V1 or V2). Panel A, N1 V1; Panel B, N1 V2; Panel C, N2 V1; Panel D, N2 V2. The solid line indicates the fitted linear regression.

Table S1. Coefficients and confidence intervals of the fitted two-parameter sigmoidal regressions for visible time course of phytomer elongation in four treatments in Fig. S3.

| Parameter | Coefficients ± 95% confidence interval | | | |
| --- | --- | --- | --- | --- |
|  | N1 V1 | N1 V2 | N2 V1 | N2 V2 |
| b | 1.73 ± 0.07 | 1.90 ± 0.08 | 1.71 ± 0.05 | 1.82 ± 0.07 |
| X0 | 1.87 ± 0.07 | 1.94 ± 0.08 | 1.69 ± 0.05 | 1.73 ± 0.07 |
| R^2^ | 0.98 | 0.97 | 0.99 | 0.99 |

The equation: y = 1/(1 + exp(− (X − X_0_)/b))

Table S2. Coefficients and confidence intervals of the fitted two-parameter sigmoidal regressions for the complete time courses of phytomer elongation in four treatments in Fig. S4.

| Parameter | Coefficients ± 95% confidence interval | | | |
| --- | --- | --- | --- | --- |
|  | N1 V1 | N1 V2 | N2 V1 | N2 V2 |
| b | 1.69 ± 0.07 | 1.84 ± 0.07 | 1.79 ± 0.06 | 1.82 ± 0.07 |
| X_0_ | 1.97 ± 0.07 | 2.02 ± 0.07 | 1.61 ± 0.05 | 1.75 ± 0.07 |
| R^2^ | 0.96 | 0.96 | 0.98 | 0.97 |

The equation: y = 1/(1 + exp(− (X − X_0_)/b))

Table S3. Coefficients and confidence intervals of the fitted linear regressions between the length of a blade and the length of its preceding sheath (i.e. length of blade Nr. n+1 vs. length of sheath Nr. n) of mature phytomers in four treatments in Fig. S5.

| Parameter | Coefficients ± 95% confidence interval | | | |
| --- | --- | --- | --- | --- |
|  | N1 V1 | N1 V2 | N2 V1 | N2 V2 |
| a | 1.56 ± 0.66 | 1.86 ± 0.53 | 1.28 ± 0.65 | 1.52 ± 0.44 |
| Y_0_ | 24.50 ± 14.29 | 13.76 ± 12.54 | 27.99 ± 15.86 | 21.53 ± 10.05 |
| R^2^ | 0.30 | 0.39 | 0.17 | 0.43 |

The equation: y = aX + Y_0_
